# GitHub is an effective platform for collaborative and reproducible laboratory research

**Published:** 2025-02-10

**Authors:** Katharine Y. Chen, Maria Toro-Moreno, Arvind Rasi Subramaniam

**Affiliations:** 1 Basic Sciences Division and Computational Biology Section of the Public Health Sciences Division, Fred Hutchinson Cancer Center, Seattle, USA

## Abstract

Laboratory research is a complex, collaborative process that involves several stages, including hypothesis formulation, experimental design, data generation and analysis, and manuscript writing. Although reproducibility and data sharing are increasingly prioritized at the publication stage, integrating these principles at earlier stages of laboratory research has been hampered by the lack of broadly applicable solutions. Here, we propose that the workflow used in modern software development offers a robust framework for enhancing reproducibility and collaboration in laboratory research. In particular, we show that GitHub, a platform widely used for collaborative software projects, can be effectively adapted to organize and document all aspects of a research project’s lifecycle in a molecular biology laboratory. We outline a three-step approach for incorporating the GitHub ecosystem into laboratory research workflows: 1. designing and organizing experiments using issues and project boards, 2. documenting experiments and data analyses with a version control system, and 3. ensuring reproducible software environments for data analyses and writing tasks with containerized packages. The versatility, scalability, and affordability of this approach make it suitable for various scenarios, ranging from small research groups to large, cross-institutional collaborations. Adopting this framework from a project’s outset can increase the efficiency and fidelity of knowledge transfer within and across research laboratories. An example GitHub repository based on this approach is available at https://github.com/rasilab/github_demo and a template repository that can be copied is available at https://github.com/rasilab/github_template.

## Introduction

Scientific progress is contingent on the ability to reproduce and build upon previous findings. To promote reproducibility of published studies, journals and funding agencies increasingly require researchers to make their data and analysis methods available in public repositories^1–3^. In parallel, data-intensive fields such as machine learning, computational biology, and ecology have developed workflows and tools that facilitate computational reproducibility^4–7^. Reproducibility is also a critical element of collaborative research, where multiple researchers need to share and build upon each other’s work. Indeed, many reproducibility standards and tools have been developed in the context of large-scale, cross-institutional collaborations^8–12^.

Compared to computational research, reproducibility and collaboration are less emphasized during the course of laboratory research, especially in the context of small research groups. This is perhaps because projects within a laboratory are often executed by individuals, and are rarely framed as collaborative efforts. However, viewed from a broader perspective, collaboration is an integral and ubiquitous feature of even small laboratory research groups. Group members discuss ideas, exchange protocols, and share reagents and data on a daily basis. Trainees collaborate with group leaders in hypothesis formulation, data interpretation, literature review, and manuscript writing. Most importantly, individuals “collaborate” with their future selves by analyzing and building on their own results, and also with future lab members who extend their work after they have left the lab. Many publicized instances of post-publication irreproducibility arise from the inability of either the same scientist at a later time or a different scientist within the same laboratory to replicate previous results^13,14^. Thus, workflows that improve scientific documentation and collaboration within laboratory groups from the outset of a project can enhance reproducibility of published studies across the wider research community.

While much effort has been devoted to improving reproducibility during data analysis and post-publication stages of research, these do not address earlier critical stages of laboratory research. Even in a ‘wet’ lab where experiments are physically performed, researchers spend significant time and resources on literature review, hypothesis formulation, experimental design, in addition to the generation, visualization and interpretation of data. Nevertheless, few tools and workflows exist to document and share these activities in a structured and reproducible manner. Lab notebooks are the most common media used to document laboratory research, but they are typically only used for recording methods and data. An NIH handbook on lab notebooks, for example, explicitly discourages the inclusion of speculative ideas or informal conversations^15^, despite the recognition that these are often the source of scientific breakthroughs. Electronic lab notebooks (ELNs), despite their popularity, are stored in proprietary formats, incur a recurrent cost, tend to become defunct over time, and have poor interoperability with each other^16^. Cloud-based tools like Google Docs, Dropbox, and Sharepoint allow sharing of data and documents, but do not provide a structured way to track changes over time or record project-related communication. Email and messaging tools such as Slack and Microsoft Teams facilitate informal discussion of ideas and data, but these are poorly suited for organizing data and discussion in a reproducible manner. Thus, it is increasingly common for data, experiment details, analysis code, and project communication to be fragmented across multiple tools within laboratory research groups.

**Figure 1:**
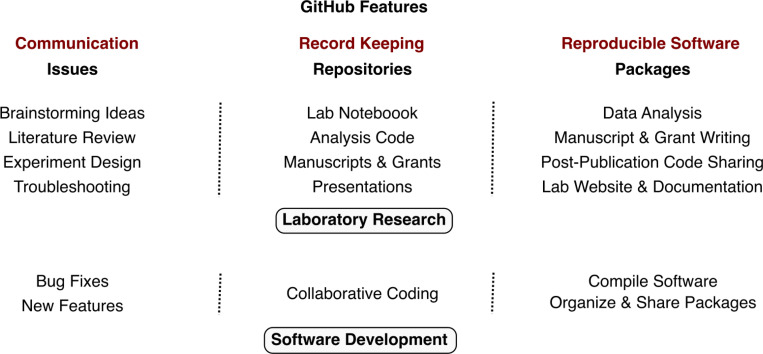


The process of software development bears several similarities to activities in laboratory research (Figure 1). Developing software involves understanding the state-of-the-art solutions to a problem, formulating a hypothesis for improving existing approaches, breaking down the hypothesis into smaller testable features, writing code to implement features, analyzing the outcome of each feature development, and troubleshooting and fixing errors when necessary. Software development occurs in groups of all sizes ranging from a single developer to thousands of contributors, often distributed across different timezones, working on a common project. The necessity to document and share all stages of software development between contributors has led to the emergence of mature tools and workflows that facilitate reproducibility and collaboration. These include concepts such as issue tracking^17^, version control^18^, and containerization^19^ that have become integral to most software development projects.

Many of the common workflows associated with software development are implemented in GitHub, a popular cloud-based platform used by over 100 million developers worldwide and 90% of Fortune 100 companies^20,21^. GitHub repositories are highly scalable, with both small projects built by single developers and large projects with over 2,000 contributors^22^. In the scientific community, GitHub is used to share data analysis workflows after publication^23,24^, develop and share computational tools^25^, perform individual record keeping^10,26,27^, conduct open science projects^11,12^, and collaboratively write manuscripts^10,28^. However, how the standard workflows and rich features of GitHub (Figure 1) can be adapted to improve reproducibility and collaboration within a traditional laboratory research group has not been explored.

**Figure 2:**
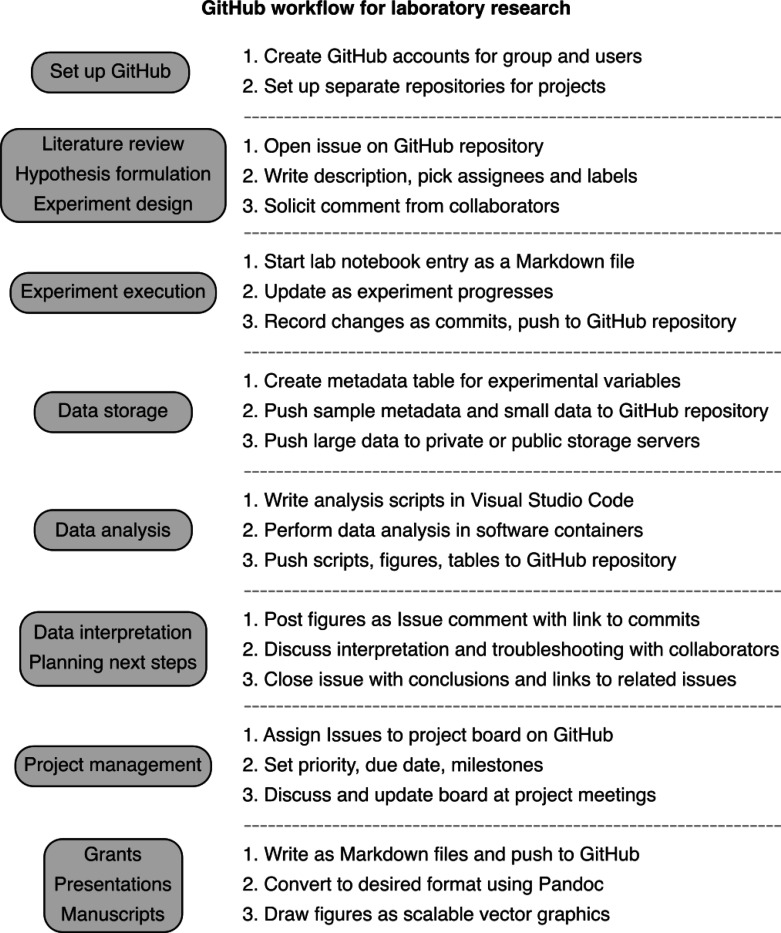


Here, we aim to provide a practical demonstration of GitHub’s use in the context of a laboratory research group centered around molecular biology experiments. Our goal is to show how GitHub provides an intuitive and structured framework to organize and document all aspects of a research project’s lifecycle, including literature review, hypothesis formulation, experiment design, lab work, data analysis, and manuscript and grant writing (Figure 2). Like many academic researchers, we started using GitHub for version control of data analysis scripts. Over the past nine years, we have expanded our use of GitHub to document all aspects of research in our laboratory, and manage collaborations both within our institution and with external collaborators. None of us had any formal training in software development or the use of GitHub. We arrived at our current workflow through trial and error, tutorials on the internet, and by seeking help from more experienced users within and outside our lab. We find that starting a project with this framework enables us to take full advantage of GitHub’s many features, though adopting this workflow at any stage of a project can still be beneficial.

### Set up GitHub for laboratory research

**Figure 3:**
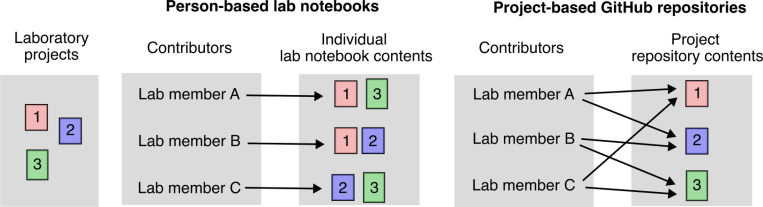


GitHub is centered around the concept of repositories, which can be thought of as cloud-based folders that contain all files and documentation related to a specific research project. While a single GitHub repository can be used to record all work by a single user, akin to a traditional lab notebook, organizing repositories based on projects provides better structure for collaborative research (Figure 3). Repositories have a unique name, which is often the project name, and a standardized URL in the format https://github.com/GROUP_NAME/PROJECT_NAME (for example, https://github.com/rasilab/ribosome_collisions_yeast). Each GitHub repository’s content can also be edited on a local computer, allowing users to work offline and synchronize with the cloud when they connect to the internet. To edit repository files locally, we typically use Visual Studio Code, a popular open source editor that works seamlessly with GitHub and has extensive features for writing and data analysis.

Adopting a GitHub-based workflow within a group or a team starts with a designated administrator creating a GitHub organization. Then, each member of the group creates a GitHub account for themselves, and are made members of the GitHub organization by the administrator. Once the organization and user accounts are set up, the administrator or any group member can create a new repository for each project the group is working on. All work and communication related to each project will be recorded within the corresponding repository. A practically unlimited number of repositories can be created within a GitHub organization, and access to each repository can be controlled by the administrator. All functionalities that we describe in the following sections are currently available as part of the free GitHub plan. Research groups in educational and non-profit institutions also get free access to the GitHub Team plan, which can be useful for accessing more advanced features.

### Use issues to organize and collaborate

Experiments are the fundamental units of laboratory research projects. Yet few standards exist to conduct the design, execution, documentation, and interpretation of experiments in a structured, reproducible, and collaborative manner. Even what constitutes a single experiment is often unclear from perusing lab notebooks. Notebooks are typically chronological records of all activities by a single researcher, making it difficult to isolate individual experiments. Further, the fragmented tools for laboratory research are reflected in the fragmented nature of the experiments themselves. Design and execution steps are recorded in lab notebooks, physical samples are stored in freezers or shelves, data is kept in centralized servers, analysis scripts are backed up in personal computers, and ideas, hypotheses, and interpretation are discussed in person or over electronic messaging apps. Tracking the full trajectory of an experiment across email discussions, physical samples, data, and analysis scripts becomes a challenge, particularly for future lab members who may need to build upon the work after the lead researcher leaves the project.

The GitHub ‘issues’ feature provides an intuitive and flexible interface to organize and collaborate on all aspects of a laboratory experiment. In software projects, issues were originally used to track bugs or problems (hence the name ‘issue’), but their utility has expanded to new feature proposals, maintenance tasks, and general discussion topics^17^. Similarly, in laboratory research, we use issues not just for troubleshooting, but for organizing and discussing all aspects of research, from hypothesis formulation and experiment design to data interpretation and manuscript writing. Each issue is limited to a single topic, which focuses the ensuing discussion and resolution. In GitHub, issues are given a unique number and a URL (https://github.com/GROUP_NAME/PROJECT_NAME/issues/ISSUE_NUMBER) that provide a centralized location to track all work and discussion related to that issue. Each issue has a description field and optional commenting fields, which can be used to write, attach files, and paste images.

**Figure 4:**
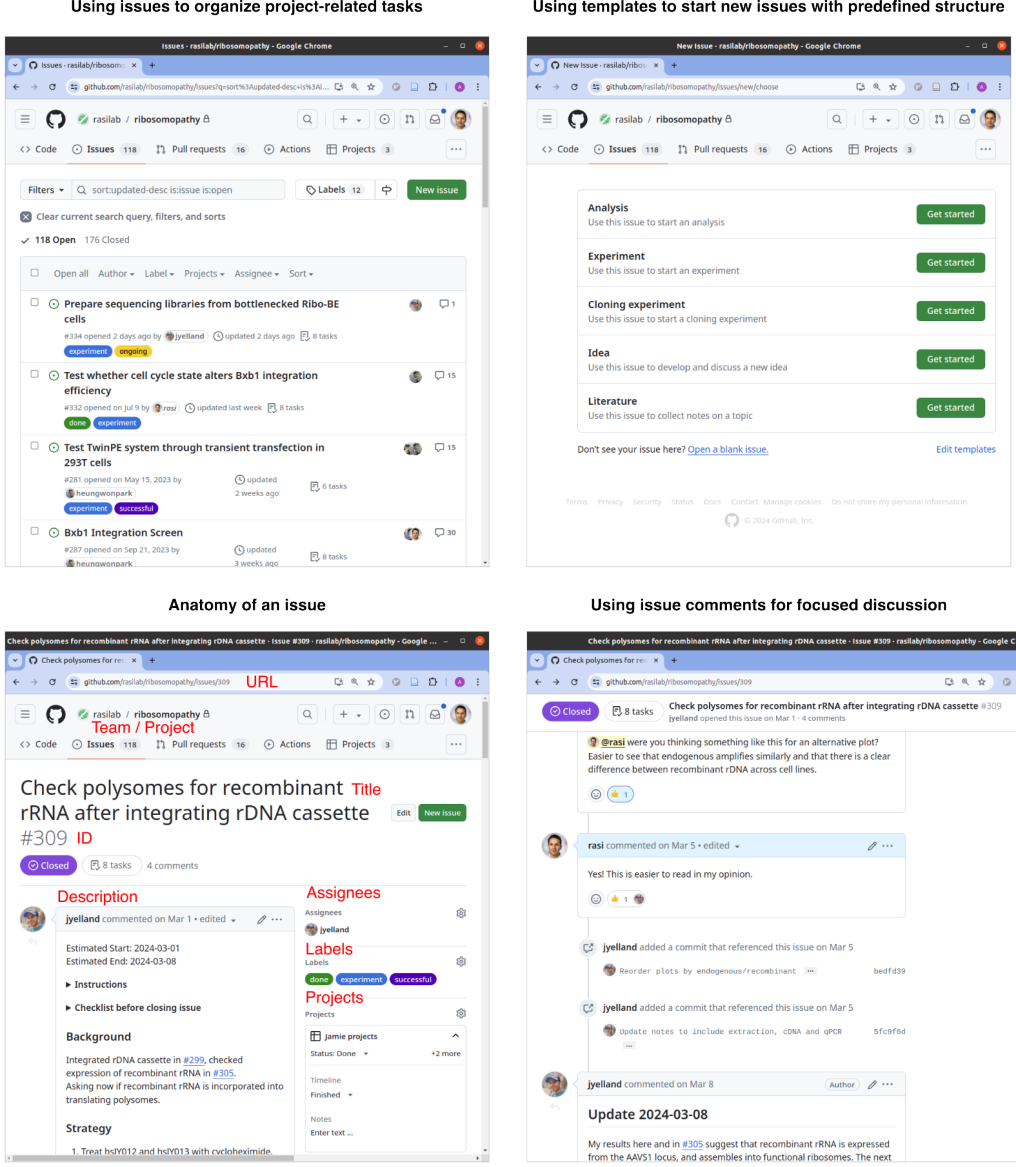


In our research group, each experiment begins with the creation of a new issue in the corresponding project repository by any of the project members (Figure 4). The issue description is used to describe the rationale and background of the experiment and the strategy for performing the experiment. Project members can discuss aspects of experimental design, provide clarification in the comments section, and update the issue description as needed. Once an experiment is started, the comments section is used to discuss troubleshooting steps, intermediate data and figures, and interpretation of results. The issue number provides a convenient way to reference the experiment across physical samples, work logs, computer file names, and discussions in other issues. For instance, we include the issue number as a prefix on the labels of sample tubes along with suffixes denoting the sample type or condition, which enables succinct and unambiguous tracking of samples by all lab members. Once an experiment is completed, the issue description is updated with key conclusions, tables, and figures, and the issue is ‘closed’. Even if the experiment proposed in an issue is paused or ultimately not pursued, there is a record of the decision-making process and the issue can be re-opened by a project member at any time. Finally, we use issues not just for experiments, but also for discussing broader ideas for projects, reviewing specific literature topics, and for collaborating on grant proposals and manuscripts. For complex experiments with multi-stage design and analysis steps, we create separate issues to document the design and analysis steps.

GitHub provides a number of features to organize and prioritize issues within a project and across projects (Figure 4). One or more ‘assignees’ can be associated with each issue to ensure that they receive notifications about any work or discussion related to the issue, and to track responsibilities. Color-coded ‘labels’ can be used to distinguish between different issue types such as ‘experiment’, ‘data analysis’, ‘literature review’, or ‘project idea’, or to indicate the state of the issue such as ‘todo’, ‘ongoing’, ‘paused’, ‘completed’, ‘abandoned’. Issues can be grouped together into ‘milestones’ to track progress towards a specific goal or deadline. For example, we use milestones to group issues that need to be completed for a figure in a manuscript or a grant. ‘Issue templates’ can also be created to standardize the format of common issue types across contributors. For example, an ‘Experiment’ issue template can include prompts to include relevant background, strategy, and conclusion in the description section and pre-populate the issue type label and common assignees such as the lead researcher on th e project. Each time a group member creates a new issue, they have the option to use one of these issue templates, which is especially useful for new or junior group members. GitHub ‘project boards’ provide a higher level visual interface to organize and prioritize issues across projects and repositories. For example, a group member can create a project board for themselves and add their own fields such as due date or priority to each issue. During project meetings, the project board can be used to quickly understand each group member’s priorities and deadlines, helping ensure that all collaborators are on the same page.

In summary, issues, a widely used feature in software development, also provide an intuitive structure to organize and collaborate across every stage of laboratory-based projects from hypothesis formulation to manuscript writing. An issue-based workflow enables group members to access and contribute to all project-related documentation and communication, regardless of the stage in which they join the project. This can be particularly useful for new lab members, who can quickly get up to speed on a project by reading through the issue descriptions and comments. Closed issues can be reopened if needed, which can be useful for revisiting old experiments or ideas. Thus, by providing a centralized location for tracking information relevant to a specific experiment, analysis, or idea, GitHub issues facilitate reproducibility and knowledge transfer during all stages of laboratory research projects.

### Use Git to store and track your work

When a researcher performs a specific experiment or data analysis task, they record the execution steps and results along the way. This record is often maintained in physical or electronic lab notebooks for wet lab experiments and as code files in computer folders for data analyses. Once a set of experiments or analyses are completed, the researcher may write a manuscript or grant proposal that summarizes the results and conclusions. Such manuscripts or grant proposals are typically written using software such as Microsoft Word or Google Docs, often in a collaborative manner with multiple authors. Since these steps can extend over several months or even years, it is frequently challenging to maintain an organized and chronological record of the contributions made by each group member, and to track the changes made to multiple files in different folders. It is common to have many copies of the same file with cryptic names like ‘manuscript_v3.docx’, ‘annotations_ARS.xls’ to indicate their provenance. While electronic lab notebooks and ‘track change’ features in word processing software can help maintain a record of changes to single files, these tools are neither designed to work across files of various types, nor to easily identify each author of overlapping changes.

Git is a version control system that records the history of file additions and modifications in a folder, and is used by over 90% of programmers worldwide to track changes to their code^21^. Git allows multiple copies of the folder to be asynchronously edited across computers, and GitHub repositories are essentially remote copies of a local folder tracked using Git. Anyone with access to a GitHub repository can download a local copy of the folder (‘clone’ in Git terminology) , add or edit file in the folder, choose which files they want to track (‘stage’), create a snapshot of the changes (‘commit’), and synchronize with the GitHub repository (‘push’). These Git features are tightly integrated into popular text editors like Visual Studio Code, which allows users to make, stage, commit, and push changes to a GitHub repository without leaving the text editor. Each commit is accompanied by a ‘commit message’ which is a short description of what changes were made since the previous commit. Importantly, the commit history of a project serves as an audit trail, recording who did what, and when.

**Figure 5:**
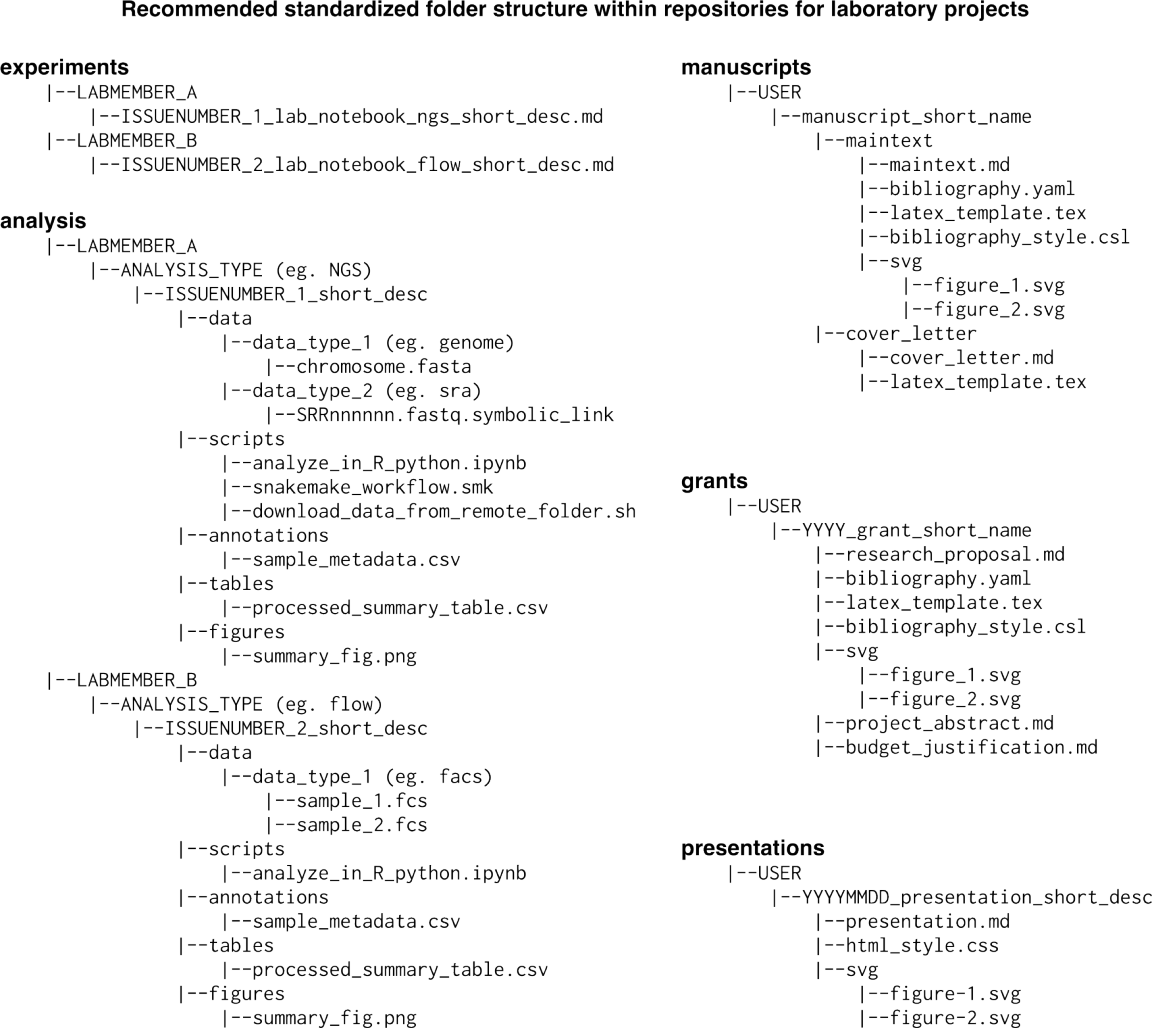


In our research group, we store all files relevant to project within a single folder on our local computers. We use Git to track changes in that folder, and synchronize it with a cloud-based GitHub repository. We write documents in plain text with lightweight Markdown syntax, as often as possible. Markdown enables focusing on content over formatting, enables all changes to be tracked by Git , and can be easily converted to other formats (PDF, DOCX, HTML) using open source software like Pandoc. Within each repository, we use standardized subfolder names for lab notebook entries, code, data, manuscripts, grants, and presentations (Figure 5). Within each of these subfolders, each project contributor creates a separate folder to record their work, even though every group member can contribute to all files in the repository. Lab notebooks entries corresponding to distinct GitHub issues are stored in separate files. We record all work pertinent to an issue in lab notebook files, similar to traditional lab notebook entries. Each lab notebook file includes the corresponding issue number in its name and a link to the issue in its contents to enable easy cross-referencing. All group members can access each other’s lab notebooks across different project repositories and participate in discussion and troubleshooting steps by commenting on the corresponding issue.

We also store data analysis materials within the GitHub project repository, which ensures that experiment logs (lab notebooks) and analysis scripts are tightly linked and easily referenced between documents. Data analysis scripts, summary tables, and visualization figures are stored in an ‘analysis’ subfolder of the parent repository, using the same hierarchical structure and naming convention as for lab notebook entries. Separate folders are created for each issue with subfolders for data, scripts, figures, tables, and sample metadata. While we store small datasets in the GitHub repository as comma- or tab-separated text files, larger datasets are stored either in private Amazon Web Services S3 folders, or in public repositories such as the Sequence Read Archive. We include short scripts to download data from their long-term storage location, which also serves as a record of the location of the data. A comma-delimited, sample metadata file is created for every dataset, following tidy principles^29^, to facilitate data analyses. Summary figures and tables are linked from the lab notebook page as a record of how the data was analyzed, and are linked from issue comments during data interpretation and troubleshooting discussions.

In addition to lab notebooks and data analyses, we use the GitHub repository to store manuscripts, grant proposals, and presentations related to the project. We write manuscripts and grant proposals as plain-text Markdown files, with one sentence per line, to enable easy tracking of changes across Git commits. Markdown files can be easily converted to DOCX, TeX, or PDF formats using Pandoc, which also provides a suite of useful features for scientific writing such as citation processing and template-based formatting to meet journal and funding agency requirements. Multiple project contributors often edit manuscript and grant proposal files in parallel, which can be readily combined using the native merge functionality of Git while preserving the full history of contributions. We prepare figures and presentation slides in the widely used text-based scalable vector graphics (SVG) format, using the powerful open-source software Inkscape. SVG files are rendered in GitHub Markdown files and web browsers, and can be processed by most commercial graphics design software. Presentations are written as Markdown files with SVG-based images and speaker notes, which can then be converted to slides in PPTX, PDF, or HTML formats using Pandoc.

In summary, the version control functionality of Git and GitHub that is widely used to track changes to software code, can also be readily used to track the work performed over the course of a project in a research laboratory, including molecular biology “wet” labs. Git repositories are drop-in replacements for lab notebooks while also providing a tightly integrated structure for data analyses, manuscript writing, grant preparation, and slide presentation tasks. By serving as a centralized location for all project-related materials, they enable contributors to reproduce and build on each other’s work. Crucially, even though we utilize GitHub for syncing repositories, project materials themselves are independent of GitHub or any other platform, which secures their long-term accessibility. Further, by providing a chronological and transparent audit of all project-related contributions, Git repositories incentivize collaboration between group members, and unambiguously indicate individual contributions during manuscript preparation and publication.

### Use containers for coding and writing tasks

Reproducibility and collaboration within a group critically depend on the ability of members to run each other’s data analyses and obtain the same results. This allows members to build on each other’s work, troubleshoot issues, and reproduce results for manuscript writing and grant preparation. However, software environments are difficult to replicate, especially when they involve multiple programming languages, packages, and dependencies, as is common for complex data analysis workflows in molecular biology. Typical challenges that group members and future collaborators face when attempting to rerun months- or years-old analysis workflows include deprecated syntax, incompatible package versions, and broken dependencies. Additionally, collaborative writing tasks such as manuscript or grant preparation require a specific set of software tools to produce the final document, which can be difficult to replicate across different operating systems and software versions. The time and effort required to troubleshoot software incompatibility issues can be substantial, and can lead to the abandonment of the task altogether.

The problem of replicating data analysis workflows and software environments is a common challenge in software development as well as in laboratory research. Computational researchers have long recognized this problem and have relied on tools such as the Conda package manager^30^, the Snakemake workflow manager^31^, and Docker containers to address it^6^. Containers are encapsulated, self-sufficient units that contain all the software needed to run an analysis, and can be shared and run on any computer that supports the container runtime environment. Public container registries, like Docker Hub or Biocontainers^32^, provide reproducibly-created containers, which can be used in data analysis workflows without the need to install any software. However, laboratory research groups have been slow to adopt these software reproducibility tools, which are often perceived as too complex or time-consuming to learn and use.

**Figure 6:**
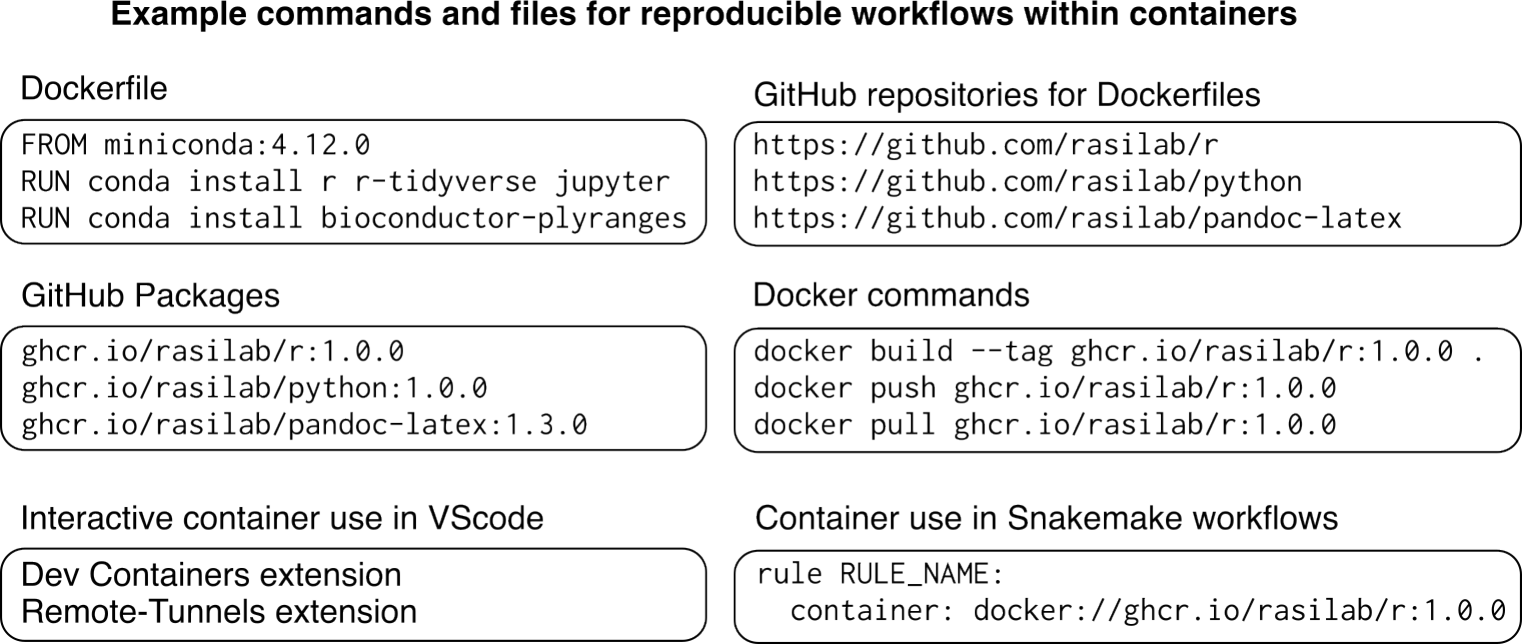


In our research group, we use software containers to perform all data analyses and writing tasks in reproducible software environments. We take advantage of the Packages feature of GitHub to host our containers in a centralized location (https://github.com/orgs/rasilab/packages) that is free to use and publicly accessible. Each container in our group’s GitHub Packages collection is linked to a dedicated GitHub repository to store the plain text recipe, called a Dockerfile, for creating that container (Figure 6). We have created a few general purpose containers with R, Python, and Pandoc software that we routinely use for data analysis and writing tasks in our group. Occasionally, we also create new containers from scratch, or modify an existing container from a public container registry to include a specific software package that is needed for a specialized analysis. We use semantic versioning to tag each container and its associated GitHub repository, which allows us to unambiguously identify its contents and use the same container in our data analysis workflows.

Our group uses containers in several ways for interactive data analyses, writing tasks, and complex bioinformatic workflows (Figure 6). Containers in our group’s GitHub Packages can also be used by external collaborators and readers of our published manuscripts to reproduce data analyses. For interactive analyses on a local computer with the Docker runtime environment, one of the general purpose containers from our group’s GitHub Packages registry can be copied (‘pulled’) to the local computer. Then the user can either access the container through the Remote-Containers extension in the Visual Studio Code editor, or run the container in a terminal window. Running containers can be used to convert Markdown files to other formats using Pandoc, or to run R or Python scripts for data analyses. In shared computing environments such as high-performance computing clusters, containers can be downloaded to a shared location and run using the Apptainer (Singularity) runtime environment. Apptainer containers in remote computing environments can be used in workflow management tools, like Snakemake, to run multi-step computational analyses, or accessed from a personal computer using the Remote-Tunnels extension in the Visual Studio Code editor for interactive analyses.

In summary, containers, which are widely used in software development and computational research, facilitate reproducible and collaborative data analysis and writing within laboratory research groups. GitHub Packages provide a centralized location for groups to store their frequently used software environments as Docker containers, and share them within the group and with the external scientific community. Once containers are set up and optimized for a group’s common workflows by an experienced group member or a bioinformatician, they can be used by all group members, far into the future, without change or detailed know-how. In our experience, containers are particularly useful for new members to get up to speed on our group’s data analysis and writing workflows without struggling to replicate the necessary software environments.

## Discussion

In this practical guide, we have described our group’s approach for tracking all stages of laboratory research from idea generation and experimental design, to data analysis and manuscript writing. Our approach is motivated by the recognition that established software development practices provide a concrete framework for addressing the reproducibility and collaboration challenges faced by laboratory research groups. We have adopted widely used features from software development workflows, such as issues, version control, and containers, and adapted them to the specific needs of a molecular biology laboratory. We have illustrated our approach using the GitHub platform, but other platforms such as GitLab and Bitbucket offer similar functionalities.

We recognize that adopting the approach outlined here can involve a steep learning curve, especially for laboratory research groups with limited computational experience. However, there are several benefits to using GitHub for laboratory research that we believe outweigh the initial investment of time and effort. First, Git and GitHub are widely used in both academia and industry, and thus the organization and documentation practices we describe are highly transferrable skills for trainees. Second, Git and GitHub have comprehensive and user-friendly documentation (Table 1), and a number of tutorials and forums are available online to help new users troubleshoot any issues that arise. Furthermore, these tools are so widely used that virtually any bioinformatician or bioinformatic core at an institution can help new teams set up and troubleshoot their GitHub workflow. Third, the workflow and features described here are highly modular. Therefore, teams can incrementally adopt them, while still deriving benefits to their overall research productivity. Finally, the approach described here costs nothing to implement, and can be used by any research group regardless of their size, funding level, or institutional affiliation.

While this guide covers the core functionalities of Git and GitHub for laboratory research, there are additional features that can further enhance collaboration and productivity. For instance, Git branching allows finer control over collaborative data analysis and writing across large teams, by allowing parallel development of different threads of ideas while retaining the history. GitHub Actions can enable the creation of automated workflows for repetitive tasks, such as updating lab website and documentation when changes are pushed to a GitHub repository. Cloud-based containers, such as GitHub Codespaces, can enable groups to perform most of their analysis and writing tasks from within a web browser without the need to install any software on their local computer. Wiki and Discussions features in GitHub allow documentation of protocols and open-ended conversations that are outside the scope of specific projects. These features, while beyond the scope of this introductory guide, can be adopted by laboratory research groups as they become more comfortable with Git and GitHub.

The organizational approach described here is tailored to the lifecycle of a conventional laboratory research project from idea generation to manuscript writing. Nevertheless, this workflow offers rich possibilities for a more reproducible and collaborative research enterprise at the institutional and community levels. For instance, GitHub issues can be used after manuscript publication to handle reagent requests and answer protocol-related questions, thus providing a centralized location for community feedback and engagement. Institutions can provide backup and support for GitHub repositories, thereby ensuring that the research record is preserved even if the original research group is no longer active or associated with the institution. With public GitHub repositories, community experts can contribute ideas and feedback during the research process, and their contributions will be visible in the repository history and issue comments. The GitHub repository itself can serve as a living manuscript, with GitHub releases or tags constituting different versions of the manuscript as it evolves over time. Thus, the approach outlined here could potentially accelerate the pace of scientific discovery by enabling faster dissemination of results and fostering more collaboration opportunities.

## Figures and Tables

**Table 1:** Guides to resources mentioned in this paper

Resource	URL

GitHub	https://docs.github.com/get-started
Markdown	https://www.markdownguide.org/getting-started
Visual Studio Code	https://code.visualstudio.com/docs
Docker	https://docs.docker.com
Git	https://swcarpentry.github.io/git-novice/
Pandoc	https://pandoc.org/MANUAL.html
Semantic versioning	https://semver.org
